# An Unusual Case of Thrombocytopenia and Renal Failure Revealing Malignancy-Associated Thrombotic Microangiopathy

**DOI:** 10.7759/cureus.111732

**Published:** 2026-06-29

**Authors:** Samuel Schwartz, Brendan Masi, Abraham E Libman, Roxana Lazarescu

**Affiliations:** 1 Medicine, Touro College of Osteopathic Medicine, New York, USA; 2 Internal Medicine, Wyckoff Heights Medical Center, New York, USA

**Keywords:** acute kidney injury, adamts13, hemodialysis, malignancy-associated tma, microangiopathic hemolytic anemia, renal biopsy, secondary tma, squamous cell carcinoma, thrombocytopenia, thrombotic microangiopathy

## Abstract

Thrombotic microangiopathy (TMA) is a process characterized by thrombocytopenia, microangiopathic hemolytic anemia, and end-organ injury. Malignancy-associated TMA is uncommon and may resemble primary TMA syndromes, particularly when renal dysfunction predominates and peripheral smear findings are limited. We present a case of biopsy-confirmed renal TMA in the setting of newly diagnosed metastatic squamous cell carcinoma (SCC) following significant diagnostic uncertainty.

A 59-year-old man with insulin-dependent diabetes mellitus presented with worsening headache, neck pain, dysphagia, shortness of breath, poor oral intake, and significant weight loss. Initial evaluation demonstrated acute hypoxic respiratory failure, progressive renal failure, thrombocytopenia, and a newly identified right-sided neck mass. Laboratory studies showed anemia, elevated lactate dehydrogenase, low haptoglobin, and reticulocytosis, raising concern for TMA. Peripheral smear showed few schistocytes, while ADAMTS13 (a disintegrin and metalloproteinase with thrombospondin type 1 motif member 13) activity was reduced but not severely deficient. Autoimmune and complement studies were unrevealing.

Renal function progressively worsened, requiring initiation of hemodialysis. Renal biopsy demonstrated acute and chronic TMA with chronic vascular changes. Additional otolaryngologic evaluation confirmed invasive, moderately differentiated nonkeratinizing SCC of the uvula with metastatic cervical disease. Given the biopsy findings, lack of severe ADAMTS13 deficiency, unrevealing autoimmune workup, and overall clinical course, malignancy-associated TMA was favored over primary thrombotic thrombocytopenic purpura and other secondary causes. Renal TMA in patients with newly diagnosed malignancy may present with a broad and overlapping differential diagnosis. In this case, renal biopsy clarified this mechanism, as the limited peripheral smear findings and multiple overlapping causes of secondary TMA had posed significant diagnostic challenges.

## Introduction

Thrombotic microangiopathy (TMA) encompasses a spectrum of disorders driven by microvascular thrombosis, clinically manifesting as consumptive thrombocytopenia, microangiopathic hemolytic anemia (MAHA), and end-organ injury. 1 Primary TMAs have well-established diagnostic and treatment pathways. For example, thrombotic thrombocytopenic purpura (TTP) is classically associated with severe deficiency of a disintegrin and metalloproteinase with thrombospondin type 1 motif member 13 (ADAMTS13) and is managed with therapeutic plasma exchange [1,2]. Secondary TMA may occur in association with severe hypertension, autoimmune disease, infection, medications, pregnancy, or malignancy, and is often more difficult to classify [3]. Malignancy-associated TMA can be particularly misleading because cancer-driven endothelial injury and coagulation activation may clinically mimic primary TMA syndromes or hypertensive emergencies [4]. Unlike primary TMA, malignancy-associated TMA does not reliably respond to standard interventions such as plasma exchange, making rapid identification of the underlying malignancy central to both diagnosis and management [1,4].

Cancer-related MAHA is most often reported in advanced malignancies. In a review of 168 reported cases, Lechner and Obermeier found that 91.8% occurred in patients with metastatic disease, most commonly involving gastric, breast, prostate, and lung cancers. However, squamous cell carcinoma (SCC) is less prominently represented among reported cases, which may make SCC-associated TMA relatively difficult to recognize [5].

Peripheral smear findings may also be misleading in renal-predominant presentations of TMA. Few or absent schistocytes do not exclude TMA when renal injury predominates, supporting the role of renal biopsy as an important diagnostic tool in selected cases [6,7]. Malignancy-associated TMA may also overlap clinically with hypertensive TMA and other secondary microangiopathic processes [4]. We present the case of a patient with metastatic SCC and biopsy-proven renal TMA in whom renal biopsy was central to the etiologic evaluation of progressive renal failure.

## Case presentation

A 59-year-old man with a past medical history of diabetes mellitus requiring insulin presented to the emergency department with three weeks of worsening headache, neck pain, and shortness of breath. During the same period, he developed abdominal pain, dysphagia with food aversion, and a 30- to 40-pound unintentional weight loss. Three days prior to admission, biopsy of a right-sided neck mass revealed a metastatic keratinizing SCC. On arrival, his blood pressure was 169/101 mmHg, and he required high-flow oxygen for acute hypoxic respiratory failure. Initial laboratory studies (Table 1) demonstrated renal dysfunction, hyponatremia, anemia, and thrombocytopenia. Reference ranges are shown in brackets when available from the hospital laboratory record. Initial/early values reflect admission or earliest available inpatient values when serial testing was available. Peak/nadir or diagnostic values reflect the most clinically relevant abnormality or one-time diagnostic result during hospitalization. Latest values reflect the latest verified value identified in the available hospital record or discharge summary when repeat testing was available. Dashes indicate that no serial value was applicable. “Not obtained” and “Not repeated” indicate that the value was unavailable for that point in time.

**Table 1:** Laboratory findings supporting TMA and evaluation of alternative etiologies ADAMTS13, a disintegrin and metalloproteinase with thrombospondin type 1 motif member 13; aPTT, activated partial thromboplastin time; BUN, blood urea nitrogen; INR, international normalized ratio; LDH, lactate dehydrogenase; PT, prothrombin time; TMA, thrombotic microangiopathy

Laboratory value (reference range)	Initial/early value	Peak/nadir or diagnostic value	Latest verified value
Renal and metabolic findings			
Creatinine (0.55–1.30 mg/dL)	2.6	6.88, peak	6.88
BUN (7–18 mg/dL)	37	83, peak	66
Sodium (136–145 mmol/L)	120	120, nadir	131
Hematologic and hemolysis markers			
Hemoglobin (13.5–18.0 g/dL)	10.5	8.5, nadir	8.6
Platelets (130–400 × 10³/μL)	57–66	57, nadir	118
LDH (84–246 U/L)	571	571, peak	334
Haptoglobin (34–200 mg/dL)	<20	<20, nadir	<20
Reticulocyte count (0.5–2.0%)	8.7	8.9, peak	2.9
Peripheral smear	Rare spherocytes; few/slight schistocytes	-	-
Evaluation of alternative TMA etiologies			
Direct antiglobulin (Coombs) test	Negative	-	-
PT/INR (PT 10.2–13.3 sec; INR 0.88–1.13)	PT 11.2; INR 0.98	PT 12.7; INR 1.11	PT 11.8; INR 1.04
aPTT (27.0–37.0 sec)	32.4	35.6	32.2
Fibrinogen (250–500 mg/dL)	Not obtained	540	Not repeated
ADAMTS13 activity (0.68–1.63 IU/mL)	Not obtained	0.50	Not repeated

Cardiopulmonary evaluation showed pulmonary edema and mildly reduced left ventricular systolic function, with a left ventricular ejection fraction of 45%; these findings were managed supportively. Non-contrast magnetic resonance imaging (MRI) of the neck on hospital day 2 further characterized the known right-sided cervical mass, measuring 4.9 × 3.5 × 7.6 cm, extending within the right carotid space from the carotid bifurcation to the skull base, with involvement of adjacent soft tissues (Figure 1). Laryngoscopy on hospital day 9 confirmed right vocal cord paralysis, though the airway remained patent.

**Figure 1:**
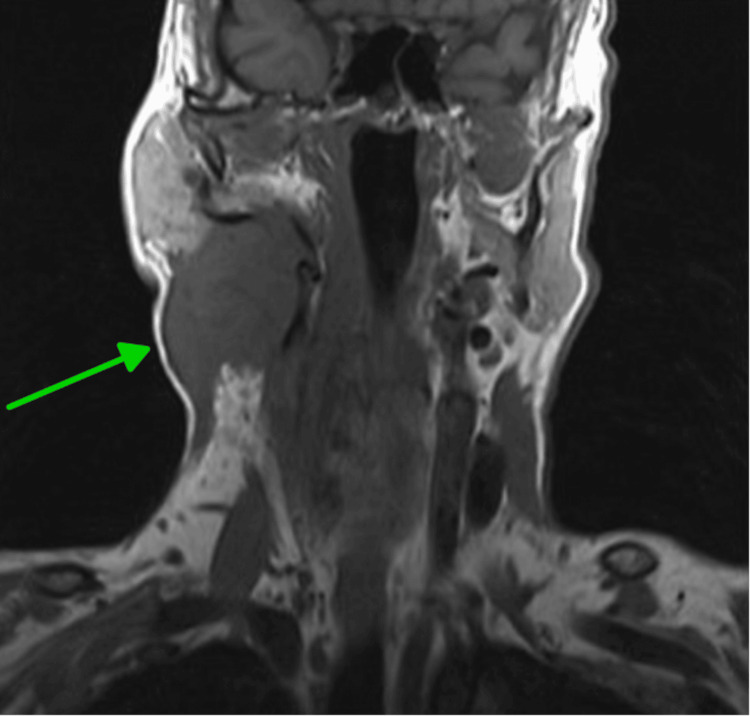
Non-contrast MRI of the neck Non-contrast MRI of the neck demonstrating a 4.9 × 3.5 × 7.6 cm right carotid space mass extending from the carotid bifurcation to the skull base. The arrow indicates the right-sided cervical mass. Evaluation was limited by the absence of intravenous contrast. Biopsy of the right neck mass performed prior to admission confirmed metastatic squamous cell carcinoma. MRI, magnetic resonance imaging

Although the peripheral blood smear showed rare spherocytes and few schistocytes, the remaining hematologic studies raised concern for a microangiopathic process. Hemolysis markers supported this concern, including haptoglobin <20 mg/dL, lactate dehydrogenase (LDH) peaking at 571 U/L, and reticulocytosis peaking at 8.9% (Table 1). Coagulation studies, including prothrombin time/international normalized ratio (PT/INR) and activated partial thromboplastin time (aPTT), were normal, fibrinogen was 540 mg/dL, and direct antiglobulin (Coombs) testing was negative. ADAMTS13 activity was 0.50 IU/mL, above the threshold for severe deficiency, which is typically diagnostic of TTP (<0.10 IU/mL) (Table 1). Autoimmune workup was unrevealing aside from a positive antinuclear antibody (ANA), with normal complement levels and negative disease-specific serologies.

Renal function worsened during admission, and hemodialysis was started on hospital day 7 through a Shiley catheter. Figure 2 shows a renal biopsy that was performed on hospital day 10 under computed tomography (CT) guidance. Pathology demonstrated acute and chronic TMA with chronic vascular changes, including severe arterial and arteriolar sclerosis and tubular atrophy involving approximately 30% of the cortex. Bone marrow biopsy performed on the same day showed no evidence of acute leukemia, lymphoma, increased blasts, or a definitive myeloid neoplasm. Platelet count and LDH improved with supportive management and dialysis, and plasmapheresis was not pursued.

**Figure 2:**
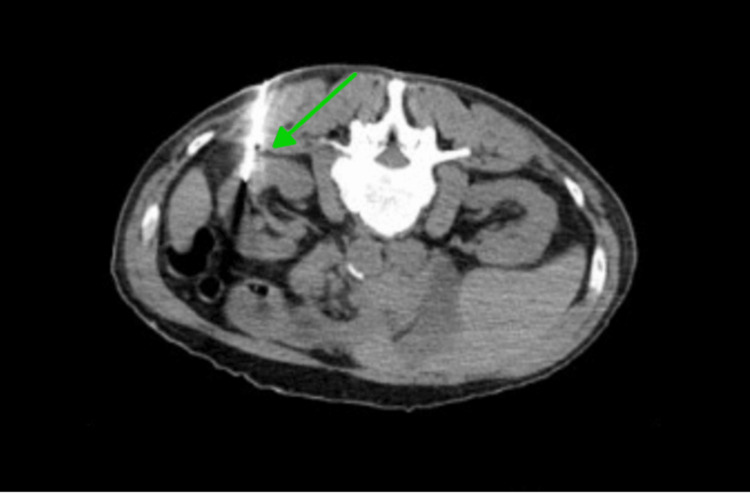
CT-guided left renal core biopsy Axial non-contrast CT scan of the abdomen with the patient in a prone position. The arrow indicates the left kidney, with the percutaneous core biopsy needle visibly entering the renal cortex. Subsequent pathology demonstrated both acute and chronic thrombotic microangiopathy with prominent arterial and glomerular capillary wall changes consistent with endothelial injury and repair. Additional chronic findings included severe arterial and arteriolar sclerosis, global glomerulosclerosis, and tubular atrophy involving approximately 30% of the cortex. The overall pathologic findings were considered most consistent with thrombotic microangiopathy. CT, computed tomography

Additional otolaryngologic biopsies were performed on hospital day 13. Pathology demonstrated invasive, moderately differentiated nonkeratinizing SCC of the uvula and SCC in situ of the left aryepiglottic fold. These findings were discussed with the treating team on hospital day 15, and given the newly diagnosed metastatic SCC, renal TMA confirmed by biopsy, and lack of findings supporting classic TTP or hematologic malignancy, malignancy-associated TMA was favored, with uncontrolled hypertension considered a contributing factor.

A tunneled dialysis catheter (PermCath) was placed in the right internal jugular vein on hospital day 15 for continued hemodialysis, and captopril 12.5 mg daily was started for blood pressure control. Given the anatomic complexity of the cervical mass and its proximity to the carotid artery, definitive surgical management was deferred at the admitting hospital, and referral was arranged with a tertiary head and neck surgeon for oncologic management. At discharge on hospital day 21, blood pressure had improved to 122/68 mmHg, sodium increased to 131 mmol/L, and platelet count improved to 118 × 10³/μL. The patient was discharged with a plan for outpatient hemodialysis, home oxygen therapy, and tertiary head and neck surgical follow-up. The patient’s hospital course and key diagnostic events are summarized in Figure 3.

**Figure 3:**
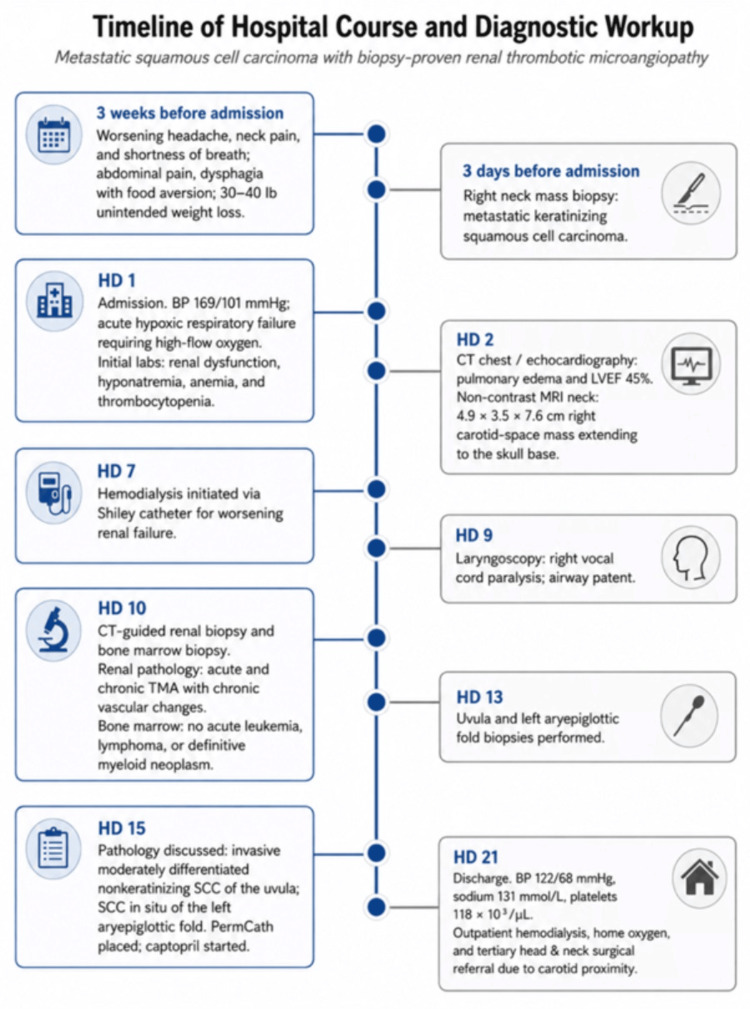
Hospital course and diagnostic workup timeline The timeline summarizes symptom development before admission, pre-admission biopsy of the right neck mass, inpatient cardiopulmonary and oncologic evaluation, initiation of hemodialysis, renal and bone marrow biopsies, additional otolaryngologic biopsies, and discharge planning with outpatient hemodialysis and tertiary head and neck surgical follow-up. BP, blood pressure; CT, computed tomography; HD, hospital day; LVEF, left ventricular ejection fraction; SCC, squamous cell carcinoma; TMA, thrombotic microangiopathy Figure created by the authors using Microsoft PowerPoint.

## Discussion

Distinguishing primary from secondary TMA presented a considerable challenge in this case, where the patient’s presentation included severe hypertension, progressive renal failure, thrombocytopenia, hemolytic laboratory abnormalities, and newly diagnosed metastatic SCC. He presented with thrombocytopenia, worsening renal failure, elevated hemolysis markers, and severe hypertension, raising concern for several overlapping thrombotic microangiopathic processes, including TTP, atypical hemolytic uremic syndrome, hypertensive TMA, autoimmune-mediated disease, and malignancy-associated TMA. The overall clinical picture, however, became less consistent with classic primary TMA syndromes over the course of his hospital stay.

Early on, TTP was considered given the findings of thrombocytopenia, anemia, elevated LDH, low haptoglobin, and progressive renal dysfunction. Although the patient's ADAMTS13 activity was below the laboratory reference range, it remained well above the severe deficiency threshold (<10%), which otherwise would have been highly suggestive of immune-mediated TTP. Furthermore, only minimal schistocytosis was observed on peripheral smear, and gradual hematologic improvement occurred without plasmapheresis. That being said, minimal peripheral smear findings have been previously reported, even in biopsy-confirmed renal TMA [7]. While empiric plasma exchange may be considered when clinical suspicion for TTP is high, the patient's subsequent clinical course and diagnostic evaluation suggested that TTP was less likely. Because the patient had presented with unexplained cytopenias in the setting of suspected TMA, a bone marrow biopsy was performed to evaluate for an underlying hematologic process; however, it showed no evidence of hematologic malignancy. Renal biopsy then demonstrated both acute and chronic TMA with chronic vascular injury, which was central to narrowing the differential diagnosis and ultimately favoring malignancy-associated TMA.

The patient also had refractory severe hypertension during the first few weeks of his hospital stay, which likely contributed to the renal injury and further complicated the diagnostic picture. Hypertension may occur as both a cause and a consequence of TMA, and distinguishing hypertensive emergencies from secondary TMA can be difficult when renal dysfunction is advanced, such as in this case [4,5]. Chronic vascular sclerosis and arteriolar changes on biopsy supported a hypertensive component; however, these findings alone did not fully explain the patient’s concurrent thrombocytopenia and hemolytic abnormalities. Autoimmune evaluation was similarly unrevealing, as, aside from a positive ANA, laboratory results revealed normal complement levels and negative disease-specific serologies, rendering autoimmune etiologies, such as lupus, scleroderma renal crisis, and others, less likely.

The presence of metastatic uvular SCC increased suspicion for malignancy-associated TMA. Cancer-related MAHA and TMA are most commonly reported in patients with advanced metastatic disease and are thought to arise through endothelial injury, microvascular tumor involvement, inflammatory cytokine release, and dysregulated coagulation pathways [4,5]. Although adenocarcinomas are more commonly reported, SCC has also been associated with secondary TMA [4,5]. The combination of metastatic SCC, biopsy-confirmed renal TMA, and exclusion of other primary TMAs ultimately favored malignancy-associated TMA in this case.

Among other reasons, this diagnosis was significant because the management of malignancy-associated TMA differs substantially from that of primary TTP, where standard treatment would have called for urgent plasmapheresis [1,4]. In secondary TMA, however, management is generally directed toward addressing the underlying malignancy, along with supportive treatment of end-organ injury. This patient required hemodialysis for progressive renal failure, while hematologic abnormalities gradually improved with supportive care and adequate blood pressure control. The clinical course, therefore, made classic TTP less likely and further supported malignancy-associated TMA, despite its unusual presentation.

## Conclusions

Distinguishing between primary and specific secondary TMAs can be challenging, especially when renal failure, thrombocytopenia, severe hypertension, and metastatic malignancy are all present concurrently. In these settings, peripheral smear findings and routine laboratory evaluation alone may not fully elucidate the underlying process(es), particularly in renal-predominant disease. Renal biopsy may therefore play an important role when multiple competing causes of secondary TMA remain under consideration.

This case also reflects the broader diagnostic uncertainty that persists among patients with malignancy-associated TMA, especially when they exhibit overlapping oncologic, hematologic, and hypertensive features. Future research evaluating the relationship between peripheral smear findings, renal pathology, ADAMTS13 activity, and clinical outcomes in secondary TMA may help improve diagnostic accuracy and efficiency, thereby informing guidance for earlier management decisions in complex oncologic presentations.

## References

[1] George JN, Nester CM (2014). Syndromes of thrombotic microangiopathy. N Engl J Med.

[2] Joly BS, Coppo P, Veyradier A (2017). Thrombotic thrombocytopenic purpura. Blood.

[3] Palma LM, Sridharan M, Sethi S (2021). Complement in secondary thrombotic microangiopathy. Kidney Int Rep.

[4] Thomas MR, Scully M (2021). How I treat microangiopathic hemolytic anemia in patients with cancer. Blood.

[5] Lechner K, Obermeier HL (2012). Cancer-related microangiopathic hemolytic anemia: clinical and laboratory features in 168 reported cases. Medicine (Baltimore).

[6] Bhutani G, Leung N, Said SM (2022). The prevalence and clinical outcomes of microangiopathic hemolytic anemia in patients with biopsy-proven renal thrombotic microangiopathy. Am J Hematol.

[7] Maisons V, Duval A, Mesnard L (2024). Assessment of epidemiology and outcomes of adult patients with kidney-limited thrombotic microangiopathies. Kidney Int.

